# 

**Published:** 1895-05

**Authors:** 


					﻿Vol xv.
MAY, 1895.,
NO. 5.
THE
pOMEOPATHlC pHY^lClAJtf,
A Monthly Journal of Homoeopathic Materia Medica
and Clinical Medicine.
“ He it a freeman whom truth makes free, and all are slaves beside.”
“Seek the Truth: come whence it may, cost what it util.”
BDITKD AND PUBLISH KD BY
WALTER M. JAMES, M. D.
PHILADELPHIA :
ZSTo. 1231 LOCUST STREET.
x»'p?ajriy’QM::
ALFRED HEATH & CO., Solk Agknts fob Gbeat Bbitain,
lHJEbury Street, Eaton Square.
Yearly Subscription, $9.50, invariably in advance. Single numbers, 95 ets.
Inured al the Philadelphia PaM-Onoc M Seoond Claaa Mall Mainer.
				

